# MicroRNA sequence analysis identifies microRNAs associated with peri-implantitis in dogs

**DOI:** 10.1042/BSR20170768

**Published:** 2017-10-11

**Authors:** Xiaolin Wu, Xipeng Chen, Wenxiang Mi, Tingting Wu, Qinhua Gu, Hui Huang

**Affiliations:** Department of Prosthodontics, Shanghai Key Laboratory of Stomatology, Ninth People’s Hospital Affiliated to Shanghai Jiao Tong University, School of Medicine, Shanghai, China

**Keywords:** microRNA, miRNA sequence analysis, osteoclasts, peri-implantitis

## Abstract

Peri-implantitis, which is characterized by dense inflammatory infiltrates and increased osteoclast activity, can lead to alveolar bone destruction and implantation failure. miRNAs participate in the regulation of various inflammatory diseases, such as periodontitis and osteoporosis. Therefore, the present study aimed to investigate the differential expression of miRNAs in canine peri-implantitis and to explore the functions of their target genes. An miRNA sequence analysis was used to identify differentially expressed miRNAs in peri-implantitis. Under the criteria of a fold-change >1.5 and *P*<0.01, 8 up-regulated and 30 down-regulated miRNAs were selected for predictions of target genes and their biological functions. Based on the results of Gene Ontology (GO) and KEGG pathway analyses, these miRNAs may fine-tune the inflammatory process in peri-implantitis through an intricate mechanism. The results of quantitative real-time PCR (qRT-PCR) revealed that let-7g, *miR-27a*, and *miR-145* may play important roles in peri-implantitis and are worth further investigation. The results of the present study provide insights into the potential biological effects of the differentially expressed miRNAs, and specific enrichment of target genes involved in the mitogen-activated protein kinase (MAPK) signaling pathway was observed. These findings highlight the intricate and specific roles of miRNAs in inflammation and osteoclastogenesis, both of which are key aspects of peri-implantitis, and thus may contribute to future investigations of the etiology, underlying mechanism, and treatment of peri-implantitis.

## Introduction

Over the past decade, implant therapy has been widely accepted and chosen as a routine procedure for the reconstruction of fully or partially edentulous individuals. However, peri-implantitis has been shown to affect long-term success following the osseointegration process and the stability of the established implants [1].

Peri-implantitis is defined as an inflammatory response surrounding implants and is characterized by an infection of the soft tissue and loss of the surrounding bone [2]. The host immune defense against bacterial challenge is responsible for the peri-implant tissue damage [3]. Lymphocytes, macrophages, and neutrophils are recruited and activated by the immune system and induce a local inflammatory response in the soft and bone tissues by producing cytokines and chemokines [4]. In particular, proinflammatory cytokines (e.g. TNF-α, IFN-γ, IL-1β, IL-6, IL-12, IL-17, and receptor activator of nuclear factor κB (NF-κB) ligand (RANKL)), anti-inflammatory cytokines (e.g. IL-4 and IL-10) and chemokines (e.g. IL-8, monocyte chemoattractant protein-1, and macrophage inflammatory protein-1α) have been suggested to be important mediators of inflammation and immunity during the pathogenesis of peri-implantitis [5–9]. Additionally, this local immune-inflammatory process is associated with disrupted bone remodeling. Osteoclastogenesis induction appears to be a major determinant of the uncoupling of bone resorption and bone formation, resulting in loss of the supporting alveolar bone and, subsequently, implant failure [10].

miRNAs are small non-coding RNA molecules that are approximately 18–22 nts long. These single-stranded molecules regulate gene expression by binding to the complementary sequences in the 3′-untranslated or coding region of the target mRNAs, leading to either blockade of translation or induction of target mRNA degradation [11,12]. Therefore, miRNAs participate in not only physiological processes within cells and tissues but also pathological processes. According to previous reports, miRNAs are specifically involved in many inflammatory and bone-related diseases, such as rheumatoid arthritis, osteoporosis, and periodontitis [13–15]. Several studies [15–17] have compared the miRNA profiles of patients with periodontitis with healthy patients, but no reports related to the miRNA profiles of patients with peri-implantitis have been published. Although the two diseases share many features, the results for periodontitis are not necessarily applicable to peri-implantitis. In fact, based on emerging evidence, peri-implantitis and periodontitis exhibit several key differences, including their histopathological and molecular characteristics [4,18]. Considering the aforementioned analysis, inflammatory miRNAs may be differentially expressed in peri-implantitis tissue compared with healthy gingival tissue.

Therefore, in the present study, we used miRNA sequencing to identify differentially expressed miRNAs in canine experimental peri-implantitis and validated the results using real-time PCR. Additionally, we ultimately determined the roles of the miRNAs in this inflammatory process by analyzing the functions of their target genes and identifying the involved pathways.

## Methods

### Animals

Six healthy adult male Labrador dogs aged 18–24 months with an approximate weight of 25 kg were used in the study. All animals were obtained from the Ninth People’s Hospital Animal Center (Shanghai, China). During the experiments, the six dogs were housed separately in kennels with 100% fresh air, an ambient temperature of 25 ± 0.1°C and humidity of 40–70%. They were fed a soft diet once daily and given free access to fresh water. All experiments in the present study were performed in accordance with an ethical permit approved by the Animal Care and Experiment Committee of the Ninth People’s Hospital affiliated to Shanghai Jiao Tong University School of Medicine, China.

### Experimental peri-implantitis

**Figure 1:**
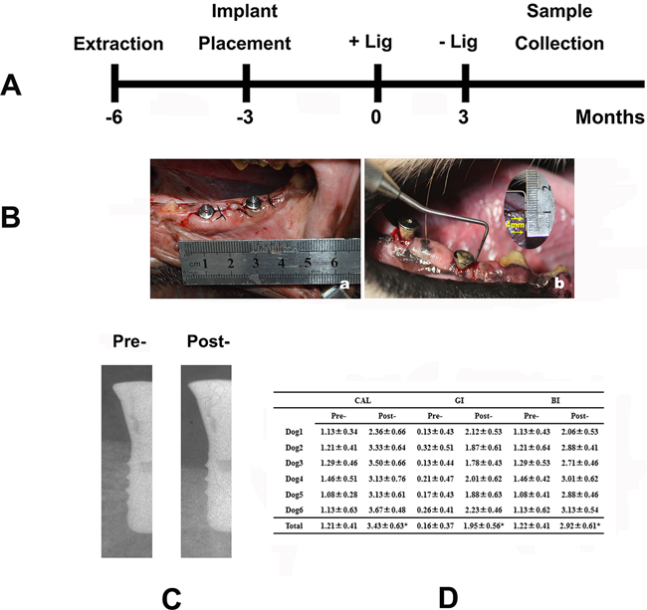
Experimental peri-implantitis (**A**) Outline of the animal experiment. (**B**) Clinical photographs: (**a**) implant installment; (**b**) 2 months after ligament placement. (**C**) Representative X-ray image of the implant before and after ligature placement. (**D**) Clinical parameters examined 2 months after the induction of inflammation, * represents *P*<0.05.

The experimental design is presented in Figure 1A. First, the bilateral mandibular fourth premolar and first molar (P4-M1) were extracted from the six dogs. Three months later, 24 (2 per side for each dog) standard ITI implants (Straumann AG, Waldenburg, Switzerland) were inserted by one experienced operator, according to the manufacturer’s recommended protocols. All implants were transmucosal and healing caps were placed. Afterward, tissue flaps were restored, and soft tissues were sutured around each implant using resorbable sutures. All surgeries were performed under general anesthesia with ketamine (10 mg/kg).

Buprenorphine HCl was administered post-operatively to all animals to relieve any pain or discomfort, and amoxicillin was used to prevent infection. The animals were allowed to move freely, were fed a soft diet, and were routinely monitored for swelling, dehiscence, and infection. Before ligature placement, plaque control was performed twice weekly using chlorhexidine and gauze.

Three months following implant insertion, peri-implantitis was induced by placing cotton floss ligatures around the implant neck, as previously described [19]. Ligatures must be closely monitored to avoid position shifts or loss. In addition, oral hygiene care was stopped after ligature insertion. The ligatures were then withdrawn 2 months after placement (Figure 1B).

Radiographic measurements (Figure 1C) and clinical examinations (Figure 1D) were performed before ligature placement and 2, 4, 6, and 8 weeks after inflammation induction to evaluate the successful establishment of the animal model. The clinical parameters examined included the clinical attachment level (CAL), gingival index (GI), and bleeding index (BI). Data were recorded from the buccal, medial, distal, and lingual sides of each implant.

### Sample collection and RNA isolation

In the present study, gingival tissues were collected from both healthy and peri-implantitis sites in all the animal subjects and were immediately frozen in liquid nitrogen. Total RNA was extracted with TRIzol (Invitrogen). The RNA concentration and purity were determined by measuring the A260/A280 ratios using the NanoDrop ND-1000 (Agilent Technologies Inc., Wilmington, DE, U.S.A.). RNA samples were immediately frozen and stored at −80°C prior to use.

### Analysis of miRNA sequences

The total RNA from each sample was used to prepare an miRNA sequencing library for that sample. This preparation process included the following steps: (i) 3′ adaptor ligation, (ii) 5′ adaptor ligation, (iii) cDNA synthesis, (iv) PCR amplification, and (v) the size-specific selection of approximately 150-bp PCR amplicons (corresponding to approximately 22-nt miRNAs). Next, the quality of each sequencing library was assessed using an Agilent 2100 Bioanalyzer (Agilent Technologies Inc., Wilmington, DE, U.S.A.) and the Agilent DNA 1000 chip kit (Agilent, part # 5067-1504). The libraries were adjusted to 10 nM before cluster generation. The samples were then denatured into ssDNA molecules, captured on Illumina flow cells, amplified *in situ* as clusters and finally sequenced for 36 cycles on an Illumina HiSeq sequencer (Illumina), according to the manufacturer’s instructions.

Single-end reads (raw reads) were harvested from the Illumina HiSeq sequencer after quality filtering. The adaptor sequences were trimmed using cutadapt software, resulting in adaptor-trimmed reads (≥15 nts). Next, the trimmed reads from all the samples were pooled and miRDeep2 software was used to predict novel miRNAs. The trimmed reads were aligned to merged pre-miRNA databases (known pre-miRNAs from miRBase plus the newly predicted pre-miRNAs) using NovoAlign software (v2.07.11), with at the most one mismatch. The number of mature miRNA-mapped tags was defined as the raw expression levels of a particular miRNA. The read counts were normalized using the tags per million approach (TPM; namely, the tag counts per million aligned miRNAs). All miRNAs with a fold-change in expression ≥1.5 or ≤ −1.5 and a *P*-value ≤0.05 were considered differentially expressed.

### Validation of sequencing results by quantitative real-time PCR

The expression levels of selected miRNAs were confirmed by quantitative real-time PCR (qRT-PCR). Briefly, the purified total RNA was reverse transcribed into cDNAs using the TaqMan MicroRNA Reverse Transcription Kit and small RNA-specific RT primers. The reactions were then incubated at 16°C for 30 min, 42°C for 30 min, and 85°C for 5 min and chilled on ice for 5 min, after which the cDNA was stored at −20°C. The qRT-PCR was performed in 20 µl reaction mixtures using the TaqMan Small RNA Assay (Applied Biosystems, Carlsbad, U.S.A.), according to the manufacturer’s instructions. U6 was used as an endogenous control to normalize the *C*_t_ values obtained for each gene. The relative expression of a miRNA compared with U6 was calculated using the 2^−ΔΔ*C*^_t_ method.

### Target prediction and functional analysis

The targets of the differentially expressed miRNAs were predicted using two different target prediction tools: TargetScan (http://www.targetscan.org/) and miRanda (http://www.microrna.org/microrna/home.do). The intersection of the two datasets was assayed based on the predicted results. To determine the potential biological functions and pathways, the top 100 targets of all differentially expressed miRNAs were further determined and subjected to Gene Ontology (GO) (http://www.geneontology.org/) and KEGG (http://www.genome.jp/kegg/) pathway analyses. The target function analysis mainly focussed on biological functions, namely, the classification of ‘Biological Processes’. Fisher’s exact test was used to calculate *P*-values. The false discovery rate (FDR) was calculated to correct the *P*-values using the Benjamini–Hochberg approach.

### Statistical analysis

Numerical data are presented as means ± S.D. The difference between means was analyzed with one-way ANOVA. Differences were considered significant when *P*<0.05. All statistical analyses were performed with the software SPSS19.0 (SPSS Inc., Chicago, IL, U.S.A.).

## Results

### Identification of differentially expressed miRNAs in canine peri-implantitis

To investigate miRNA expression in peri-implantitis, we performed miRNA sequencing using gingival tissues obtained from a canine peri-implantitis model. Based on the criteria described above (*P*<0.01 and a fold-change in expression ≥1.5 or ≤ −1.5), a total of 38 miRNAs were found to be differentially expressed (Table 1). Of these miRNAs, 8 were overexpressed in the diseased tissue compared with healthy tissue, and 30 were underexpressed.

**Table 1:** List of the top differentially expressed miRNAs between diseased and healthy gingival tissues (*P*<0.01)

miRNAs	Mature sequence	Fold-change	Regulation
(A) Top eight miRNAs with increased expression in diseased compared with healthy gingiva			
cfa-*miR-452*	AACUGUUUGCAGAGGAAACUGA	2.52	Up
cfa-*miR-375*	UUUGUUCGUUCGGCUCGCGUGA	2.43	Up
cfa-*miR-98*	UGAGGUAGUAAGUUGUAUUGUU	2.38	Up
cfa-*miR-145*	GUCCAGUUUUCCCAGGAAUCCCU	1.96	Up
cfa-let-7e	UGAGGUAGGAGGUUGUAUAGUU	1.71	Up
cfa-*miR-142*	CCCAUAAAGUAGAAAGCACUA	1.71	Up
cfa-*miR-500*	AUGCACCUGGGCAAGGAUUCU	1.70	Up
cfa-*miR-7*	UGGAAGACUAGUGAUUUUGUUGU	1.68	Up
(B) Top 30 miRNAs with decreased expression in diseased compared with healthy gingiva			
cfa-let-7g	UGAGGUAGUAGUUUGUACAGUU	−1.51	Down
cfa-*miR-486*	UCCUGUACUGAGCUGCCCCGA	−1.51	Down
cfa-*miR-152*	UCAGUGCAUGACAGAACUUGG	−1.51	Down
cfa-*miR-127*	UCGGAUCCGUCUGAGCUUGGCU	−1.55	Down
cfa-*miR-1271*	CUUGGCACCUAGUAAGCACU	−1.56	Down
cfa-*miR-101*	UACAGUACUGUGAUAACUGA	−1.57	Down
cfa-*miR-16*	UAGCAGCACGUAAAUAUUGGCG	−1.61	Down
cfa-*miR-200a*	CAUCUUACCGGACAGUGCUGGA	−1.61	Down
cfa-*miR-140*	ACCACAGGGUAGAACCACGGA	−1.67	Down
cfa-*miR-27b*	UUCACAGUGGCUAAGUUCUGC	−1.69	Down
cfa-*miR-146a*	UGAGAACUGAAUUCCAUGGGUU	−1.74	Down
cfa-*miR-204*	UUCCCUUUGUCAUCCUAUGCCU	−1.80	Down
cfa-*miR-29a*	UAGCACCAUCUGAAAUCGGUUA	−1.81	Down
cfa-*miR-26a*	UUCAAGUAAUCCAGGAUAGGCU	−1.83	Down
cfa-*miR-451*	AAACCGUUACCAUUACUGAGUU	−1.89	Down
cfa-*miR-125a*	UCCCUGAGACCCUUUAACCUGU	−1.92	Down
cfa-*miR-93*	CAAAGUGCUGUUCGUGCAGGUAG	−1.96	Down
cfa-*miR-361*	UUAUCAGAAUCUCCAGGGGUAC	−1.97	Down
cfa-*miR-27a*	UUCACAGUGGCUAAGUUCCG	−2.03	Down
cfa-*miR-532*	CAUGCCUUGAGUGUAGGACCGU	−2.09	Down
cfa-let-7c	UGAGGUAGUAGGUUGUAUGGUU	−2.11	Down
cfa-*miR-23a*	AUCACAUUGCCAGGGAUUU	−2.33	Down
cfa-*miR-211*	UUCCCUUUGUCAUCCUUUGCCU	−2.35	Down
cfa-*miR-92b*	UAUUGCACUCGUCCCGGCCUCC	−2.40	Down
cfa-*miR-342*	UCUCACACAGAAAUCGCACCCGU	−2.58	Down
cfa-*miR-340*	UUAUAAAGCAAUGAGACUGAUU	−3.04	Down
cfa-*miR-374a*	UUAUAAUACAACCUGAUAAGU	−3.09	Down
cfa-*miR-9*	UCUUUGGUUAUCUAGCUGUAUGA	−4.22	Down
cfa-*miR-429*	UAAUACUGUCUGGUAAUGCCGU	−5.58	Down
cfa-*miR-20a*	UAAAGUGCUUAUAGUGCAGGUAG	−7.52	Down

According to several previous reports, most of the low-abundance miRNAs function in inflammatory processes, including bone resorption (*miR-26a, miR-27a, miR-101*, and *miR-20a*), periodontal ligament development and mineralization (*miR-101*), inflammatory cytokine secretion (*miR-27a, miR-93, miR-451, let-7c, miR-146a*, and *miR-20a*) and endothelial dysfunction (let-7g). However, as mentioned above, although the absolute number of significantly differentially expressed low-abundance miRNAs was high, certain miRNAs were expressed at increased levels in peri-implantitis. Interestingly, nearly all the high-abundance miRNAs are known to be strongly associated with processes related to the inflammatory response and bone metabolism, such as osteoclast activity (*miR-142, miR-145, miR-375*, and let-7e), inflammation-related signaling (*miR-98*), apoptosis (*miR-142*), tissue fibrogenesis (*miR-142*), and the regulation of endothelial cells (let-7e). Thus, the differentially expressed miRNAs may be associated with the pathology of peri-implantitis.

### Confirmation of differentially expressed miRNAs by qRT-PCR

**Figure 2:**
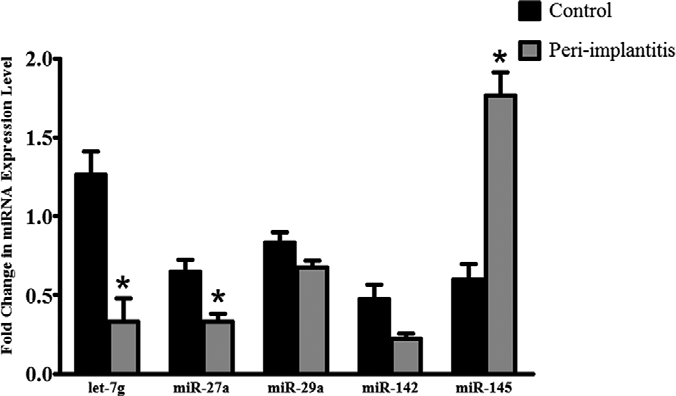
Validation of sequencing data by qRT-PCR Fold-changes in the expression of three down-regulated miRNAs (let-7g, *miR-27a*, and *miR-29a*) and two up-regulated miRNAs (*miR-142* and *miR-145*) were verified. *, statistically significant difference between the peri-implantitis and control groups, *P*<0.05.

To confirm the findings obtained by analyzing the miRNA profile, we selected five miRNAs (let-7g, *miR-27a, miR-29a, miR-142*, and *miR-145*) and measured their expression using qRT-PCR (Figure 2). The chosen miRNAs were broadly conserved and related to inflammation and included both miRNAs with relatively increased expression and miRNAs with decreased expression, as reflected by the fold-change in expression. With the exception of *miR-142*, the results for all miRNAs examined using the quantitative assay were consistent with the data obtained from the miRNA sequence analysis, further confirming that these miRNAs may participate in peri-implant infection and validating the accuracy of the sequencing approach. Amongst the four miRNAs, let-7g, *miR-27a*, and *miR-145* were significantly differentially expressed between inflamed tissues and healthy control tissues (*P*<0.05).

### Target prediction and functional annotations

In general, only a fraction of predicted targets have been confirmed to be regulated by a given miRNA in a specific tissue [20]. Therefore, only the top 100 target genes (results not shown) from overlapping results obtained using two well-established prediction tools, TargetScan and miRanda, amongst all differentially expressed miRNAs were retained and considered for further functional annotation analysis to increase the likelihood of predicting genuine miRNA targets.

**Figure 3:**
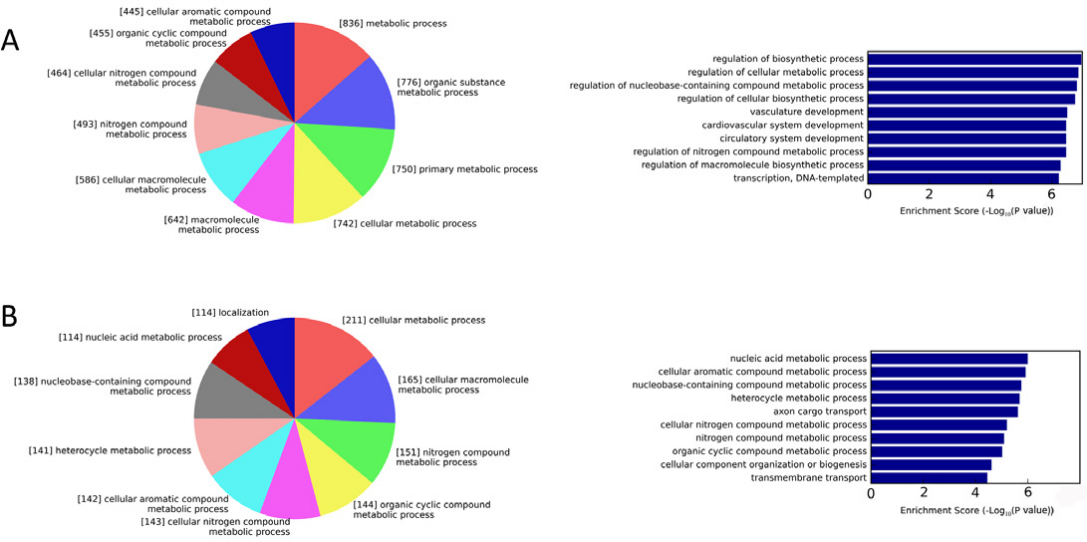
Functional annotations of target genes Biological process categories of target genes of low-abundance miRNAs (**A**) and high-abundance miRNAs (**B**) in the GO analysis. The numbers shown in each section of the pie chart represent the number of putative target genes mapped to the corresponding GO terms. Bar graphs show the −log10 (*P*-value) of each GO term.

To clearly interpret the role of the miRNAs in changes in disease development in either direction, we next performed GO and KEGG analyses for up-regulated genes (targets of the low-abundance miRNAs) and down-regulated genes (targets of the high-abundance miRNAs), respectively. GO terms with *P*<0.001 and FDR ≤25% were considered statistically significant. Regarding the biological functions associated with the predicted targets, the up-regulated genes represented 75 GO terms, amongst which regulation of macromolecule biosynthetic processes, cell adhesion, phosphorylation, angiogenesis, and the mitogen-activated protein kinase (MAPK) cascade, amongst other terms, were all correlated with active inflammation. The GO term of regulation of macromolecule biosynthetic processes has been described to be associated with macrophage differentiation [21,22], which is closely related to osteoclast precursor development [23]. Furthermore, the regulation of biosynthetic processes, regulation of cellular metabolic processes, and regulation of nucleobase-containing compound metabolic processes ranked as the top three over-represented GO terms and regulation of cellular metabolic process was associated with the greatest number of genes (Figure 3A). Correspondingly, nucleic acid metabolic processes, cellular aromatic compound metabolic processes, and nucleobase-containing compound metabolic processes were prominent amongst the enriched GO terms for down-regulated genes (Figure 3B).

**Figure 4:**
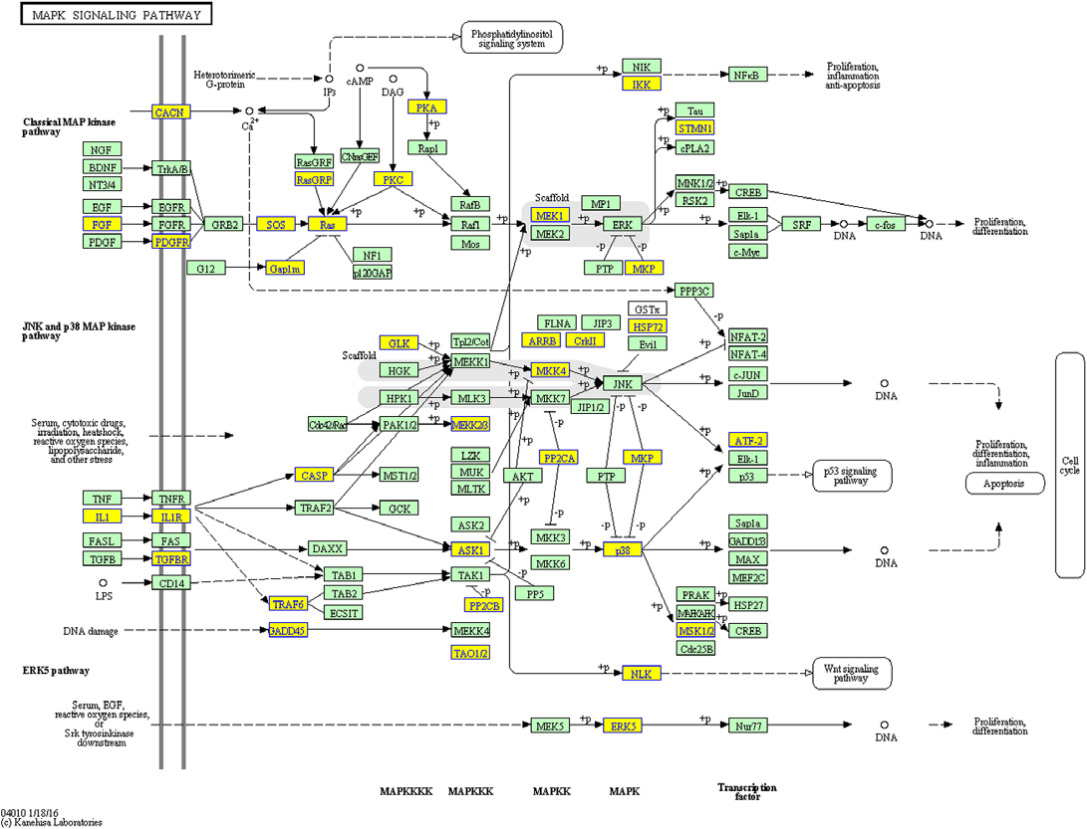
The KEGG analysis annotated the MAPK signaling pathway as significantly enriched in peri-implantitis Yellow nodes represent up-regulated genes enriched in peri-implantitis.

Regarding the pathway analysis, 14 and 38 statistically significant (*P*<0.05) terms were identified for down-regulated genes and up-regulated genes, respectively (Tables 2 and 3). Amongst the pathways for up-regulated genes, the MAPK signaling pathway (Figure 4), the NF-κB signaling pathway (Supplementary Figure S1) and the toll-like receptor signaling pathway (Supplementary Figure S2) were highly enriched in the KEGG analysis. Other pathways that are closely correlated with inflammation regulation and osteoclast activity were also statistically significant, such as the PI3K-Akt signaling pathway, the TNF signaling pathway and the TGF-β signaling pathway, indicating that miRNAs mediate the inflammatory process through their effects on intricate signaling pathways. In contrast, the KEGG pathway evaluation of down-regulated genes in peri-implantitis lesions revealed that the genes were mostly implicated in the following pathways, amongst others: phagosomes, adherens junctions, thyroid hormone signaling, and lysosomes. Phagosomes and lysosomes are both related to the digestion and removal of bacteria.

**Table 2:** Enriched KEGG pathways involving target genes of 30 low-abundance miRNAs (*P*<0.05)

Pathway ID	Definition	Hits	Total	Percent (%)	Fisher-P value
cfa05206	MiRNAs in cancer	31	139	22.30%	2.70E-05
cfa05200	Pathways in cancer	62	393	15.78%	4.29E-04
cfa04010	MAPK signaling pathway	43	252	17.06%	6.55E-04
cfa04060	Cytokine–cytokine receptor interaction	38	216	17.59%	7.35E-04
cfa04151	PI3K-Akt signaling pathway	53	334	15.87%	9.73E-04
cfa03060	Protein export	8	23	34.78%	1.55E-03
cfa04933	AGE-RAGE signaling pathway in diabetic complications	21	102	20.59%	1.57E-03
cfa04510	Focal adhesion	35	204	17.16%	1.84E-03
cfa04810	Regulation of actin cytoskeleton	36	212	16.98%	1.92E-03
cfa04974	Protein digestion and absorption	18	84	21.43%	2.08E-03
cfa04062	Chemokine signaling pathway	30	172	17.44%	2.94E-03
cfa04722	Neurotrophin signaling pathway	22	120	18.33%	5.56E-03
cfa04320	Dorsoventral axis formation	8	28	28.57%	6.14E-03
cfa05205	Proteoglycans in cancer	32	197	16.24%	6.65E-03
cfa04666	Fc γ R-mediated phagocytosis	17	88	19.32%	8.25E-03
cfa04068	FoxO signaling pathway	23	132	17.42%	8.72E-03
cfa04668	TNF signaling pathway	19	105	18.10%	1.10E-02
cfa04670	Leukocyte transendothelial migration	21	120	17.50%	1.13E-02
cfa04360	Axon guidance	22	128	17.19%	1.19E-02
cfa04550	Signaling pathways regulating pluripotency of stem cells	23	138	16.67%	1.47E-02
cfa05212	Pancreatic cancer	13	65	20.00%	1.48E-02
cfa00310	Lysine degradation	11	52	21.15%	1.60E-02
cfa04115	p53 signaling pathway	13	66	19.70%	1.67E-02
cfa04350	TGF-β signaling pathway	15	82	18.29%	2.04E-02
cfa04512	ECM–receptor interaction	15	82	18.29%	2.04E-02
cfa05222	Small cell lung cancer	15	84	17.86%	2.50E-02
cfa04919	Thyroid hormone signaling pathway	19	115	16.52%	2.72E-02
cfa02010	ABC transporters	9	43	20.93%	2.99E-02
cfa05166	HTLV-I infection	35	246	14.23%	3.26E-02
cfa04064	NF-κB signaling pathway	15	87	17.24%	3.32E-02
cfa05202	Transcriptional misregulation in cancer	24	158	15.19%	3.59E-02
cfa05220	Chronic myeloid leukemia	13	73	17.81%	3.61E-02
cfa05146	Amoebiasis	16	96	16.67%	3.77E-02
cfa04917	Prolactin signaling pathway	13	74	17.57%	3.98E-02
cfa04144	Endocytosis	35	252	13.89%	4.42E-02
cfa04620	Toll-like receptor signaling pathway	16	98	16.33%	4.44E-02
cfa04014	Ras signaling pathway	31	220	14.09%	4.74E-02
cfa00603	Glycosphingolipid biosynthesis – globo series	4	14	28.57%	4.96E-02

**Table 3:** Enriched KEGG pathways involving target genes of eight high-abundance miRNAs (*P*<0.05)

Pathway ID	Definition	Hits	Total	Percent (%)	Fisher-P value
cfa04145	Phagosome	11	141	7.80%	3.07E-03
cfa04520	Adherens junction	7	73	9.59%	5.73E-03
cfa04919	Thyroid hormone signaling pathway	9	115	7.83%	7.04E-03
cfa04142	Lysosome	9	117	7.69%	7.86E-03
cfa04320	Dorsoventral axis formation	4	28	14.29%	8.87E-03
cfa05412	Arrhythmogenic right ventricular cardiomyopathy (ARVC)	6	72	8.33%	1.97E-02
cfa05152	Tuberculosis	10	164	6.10%	2.40E-02
cfa04916	Melanogenesis	7	100	7.00%	2.89E-02
cfa05323	Rheumatoid arthritis	6	82	7.32%	3.47E-02
cfa04390	Hippo signaling pathway	9	153	5.88%	3.83E-02
cfa05414	Dilated cardiomyopathy	6	85	7.06%	4.03E-02
cfa00130	Ubiquinone and other terpenoid-quinone biosynthesis	2	11	18.18%	4.05E-02
cfa03420	Nucleotide excision repair	4	44	9.09%	4.08E-02

## Discussion

Peri-implant infection is initiated by biofilm accumulation around the implant surface. At the initial stage, peri-implant mucositis is established, wherein inflammation culminates in an acanthotic epithelium, connective tissue loss, microvascular changes, and increased infiltration of T and B cells, neutrophils, and macrophages [24]. The swift progression to peri-implantitis is characterized by increased proportions of immune cells and associated inflammatory mediators and a relatively large infiltrate that expands apically to the junctional epithelium toward the bone marrow [17,25]. Hence, peri-implant inflammation appears to be triggered by a set of consecutive events, including an immune response; cell apoptosis, proliferation, and differentiation; and immune cell adhesion and migration. However, although peri-implant infections are well described histopathologically, the molecular events that govern the development of these infections have not yet been completely characterized. Certain studies have attempted to correlate miRNAs with the pathogenesis of peri-implantitis [26]. In particular, it has been noted that *miR-146a* and *miR-499* gene polymorphisms may be genetic determinants of an increased risk of peri-implantitis in Iranians. To date, the present study is the first to examine the expression of miRNAs in peri-implantitis and to provide a foundation for further mechanistic experiments aimed at dissecting the roles of miRNAs in peri-implant tissue homeostasis and pathology.

Because high genetic heterogeneity in the human population may skew experimental results, we established an experimental peri-implantitis model in six similar dogs using the ligature induction method. A very high prevalence of periodontitis has been reported in the canine population, and the histological traits of the diseased periodontium are similar between canines and humans [27]. Moreover, the bone defects observed in ligature-induced peri-implantitis seem to resemble naturally occurring lesions in humans [28]. RNA-Seq, as a very high-throughput quantitative method, is reported to be highly accurate in quantitating gene expression levels and sensitive in quantitating genes expressed at either low or very high levels [29]. Therefore, we employed this method to obtain miRNA expression patterns in peri-implantitis by comparing inflamed peri-implant and healthy gingival tissues.

According to the RNA-Seq analysis, 8 and 30 miRNAs were up-regulated and down-regulated, respectively, using the criteria *P*≤0.01 and a fold-change in expression ≥1.5. These miRNAs are likely to promote disease or to be expressed in an attempt to down-regulate immune activation and bone resorption in peri-implantitis lesions. A well-known example of the former is let-7e, a member of the let-7 family that is evolutionarily conserved from bacteria to humans. Specifically, let-7e acts as a key regulator of bone metabolism and inflammation. For example, let-7e overexpression activates Th1 and Th17 cells and aggravates experimental autoimmune encephalomyelitis and Hashimoto’s disease by targetting IL-10 [30,31]. Furthermore, let-7e enhances the inflammatory response in endothelial cells and promotes proper differentiation of monocytes into osteoclasts by stimulating both the nuclear translocation of NF-κB and activation of the NF-κB pathway [32,33]. Immune cells activated by proinflammatory cytokines and adhesion molecules adhere to and migrate through the vessel wall and into inflammatory sites, followed by bone resorption.

Many of the miRNAs identified here are also known to have disease-suppressing functions in periodontitis and other inflammatory diseases. Two examples are *miR-27a* and *miR-146a*, which are expressed at lower levels in diseased tissues. Notably, *miR-27a* functions as a positive regulator of osteogenic differentiation via complex modulatory mechanisms [34–36]. In addition, *miR-27a* is down-regulated in macrophages following TLR2 and TLR4 stimulation and increases the expression of IL-10, one of the most important anti-inflammatory mediators. Down-regulation of *miR-27a* activates the IL-10-dependent JAK1-STAT3 pathway, likely to be as a result of increased IL-10 expression [37]. Based on these findings, we propose that the *miR-27a* down-regulation identified in the present study may act as a negative regulatory mechanism that prevents overly exuberant TLR2- and TLR4-driven inflammatory responses. In addition, tipping the balance between osteoblasts and osteoclasts toward bone resorption in peri-implantitis may also explain why *miR-27a* is down-regulated. The other miRNA, *miR-146a*, is up-regulated upon TLR activation by LPS or by proinflammatory cytokines such as IL-1β and TNF-α in an NF-κB-dependent manner [38,39]. Both IL-1β and TNF-α are important mediators of the pathogenesis of peri-implantitis [9]. IL-1 receptor associated kinase (IRAK1) and TNF receptor associated factor-6 (TRAF6) are down-regulated by *miR-146a*, thereby producing a negative feedback loop to fine-tune the immune response [40]. In contrast with most reports of *miR-146a* expression in patients with periodontitis [16,41,42], the present study showed low expression of *miR-146a* in inflamed peri-implant tissues. Therefore, we readily understand the high proportions of IL-1β and TNF-α and a more aggressive immune reaction in peri-implantitis compared with periodontitis.

To understand the functional outcome of miRNA dysregulation, we investigated the pathways and functions enriched in the target genes. In addition to processes relevant to cell proliferation and basic metabolism, two of the most enriched functions in the target genes were the regulation of macromolecule biosynthesis processes and nitrogen-containing compound metabolic processes. Macromolecule synthesis is the main housekeeping function of immune cells, and it is required for immune activation [43]; macromolecules are responsible for inducing the expression of proinflammatory cytokines [44]. Cytokines, such as TNF-α, IL-1β, IL-6, and IL-17, have been shown to directly or indirectly promote osteoclast differentiation and function by acting on cells of the osteoclast lineage or other cell types to regulate expression of the key osteoclastogenic factor RANKL and/or its inhibitor, osteoprotegerin (OPG) [45,46]. The metabolic processes for nitrogen-containing compounds mainly involve inducible nitric oxide (NO) synthase (iNOS) and NO. According to previous studies, iNOS induces NO production and is required for *Porphyromonas gingivalis* (Pg) induced alveolar bone loss in periodontal disease. Meanwhile, NO is an important element of the host defense machinery against the periodontal pathogen Pg [47,48]. Collectively, the GO results collected in the current study elucidated the role of the differentially expressed miRNAs in regulating bone destruction and the expression of immune-related genes.

RANKL, a member of the TNF superfamily, is a protein located on the membrane of osteoblasts and their precursors that recognizes its receptor, RANK, on marrow macrophages, prompting them to assume the osteoclast phenotype. Importantly, the function of these cells in osteoclast metabolism has been recognized in peri-implantitis, and RANK may be a pathological determinant of peri-implantitis [49]. TRAF proteins are recruited following RANKL and RANK binding and then regulate the transduction of signals from RANK, with subsequent activation of the NF-κB and MAPK pathways. These two signaling pathways directly regulate the expression of osteoclastic genes, such as *CTSK, MMP-9*, and *TRAP*, which regulate the formation of bone resorption pits during osteoclast differentiation [50]. Interestingly, the pathways described above, particularly the MAPK signaling pathway, are all predominantly enriched in peri-implantitis lesions. As mentioned above, the potential target genes of *miR-146a* include IRAK1 and TRAF6. These two target genes are essential signaling components of IL-1R- and TLR-mediated NF-κB and MAPK activation [51–53]. Thus, down-regulation of *miR-146a* in peri-implantitis should diminish the inhibition of IRAK1 and TRAF6 and promote activation of the NF-κB and MAPK pathways. However, researchers have not clearly determined whether *miR-146a* down-regulation promotes osteoclast differentiation in peri-implantitis. Thus, future studies are warranted to validate the function and mechanism of *miR-146a* in the osteoclastogenesis of alveolar bone marrow cells.

In the present study, we used an miRNA sequence analysis to identify the miRNAs that were differentially expressed during peri-implantitis. Bioinformatics technology provided insights into the potential biological effects and showed particular enrichment of target genes involved in the MAPK signaling pathway. These findings shed light on the regulatory mechanisms underlying peri-implantitis and may open new avenues for future research into the etiology, mechanism, and treatment of peri-implantitis. The role of certain miRNAs in peri-implantitis is currently being investigated further.

## Supporting information

## Abbreviations

FDR: false discovery rate
GO: Gene Ontology
iNOS: inducible nitric oxide synthase
IRAK1: IL-1 receptor associated kinase
MAPK: mitogen-activated protein kinase
NF-κB: nuclear factor κB
NO: nitric oxide
Pg: *Porphyromonas gingivalis*
qRT-PCR: quantitative real-time PCR
RANKL: receptor activator of NF-κB ligand
TRAF6: TNF receptor associated factor-6
TNF-α: tumor necrosis factor-α
TGF-β: transforming growth factor-β
TLR: Toll-like receptor
LPS: lipopolysaccharides
CTSK: cathepsin K
TRAP: tartrate-resistant acid phosphatase
