# Supplementary figures and images for: MicroRNA sequence analysis identifies microRNAs associated with peri-implantitis in dogs
Source: Biosci Rep. 2017 Oct 11;37(5):BSR20170768. doi: 10.1042/BSR20170768 (PMC5964874; doi:10.1042/BSR20170768)

NF- $\kappa$ B IN SIGNALING PATHWAYS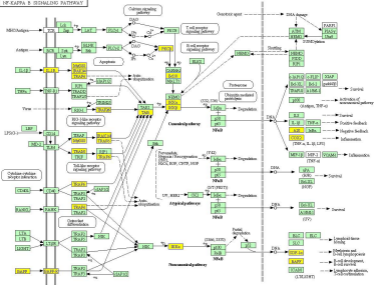

Supplement: Supplementary file 1 [file bsr-37-bsr20170768_Supp1.pdf]
